# Strain-engineered Si-doped Cs_3_Bi_2_I_9_ perovskite for high-performance MIM capacitors: a DFT study

**DOI:** 10.1039/d6ra01339b

**Published:** 2026-03-27

**Authors:** Yahaya Saadu Itas, Mayeen Uddin Khandaker, Faiza Benabdallah

**Affiliations:** a Applied Physics and Radiation Technologies Group, CCDCU, Faculty of Engineering and Technology, Sunway University 47500 Bandar Sunway Selangor Malaysia yitas@sazu.edu.ng mayeenk@sunway.edu.my; b Department of Physics, Sa'adu Zungur University Gadau Nigeria; c Department of Physics, College of Science, Korea University 145 Anam-ro Seongbuk-gu Seoul 02841 Republic of Korea; d Miyan Research Institute, International University of Business Agriculture and Technology Dhaka-1230 Bangladesh; e Department of Industrial and Systems Engineering, College of Engineering, Princess Nourah bint Abdulrahman University P.O. Box 84428 Riyadh 11671 Saudi Arabia

## Abstract

This study examines the energy storage potential of strain engineered Si doped Cs_3_Bi_2_I_9_ perovskites using density functional theory. Key electronic and electromechanical parameters—band gap, born effective charge, polarization, piezoelectricity, and leakage suppression—were evaluated for intrinsic and strained systems. Undoped Cs_3_Bi_2_I_9_ exhibits a wide 3.3 eV band gap and low polarization, making it suitable as a stable insulating material. Introducing 0.25 mol% Si narrows the band gap and introduces beneficial defects that enhance the dielectric constant and capacitance, with BEC analysis revealing strong local polarization around Si atoms and increased anisotropic stiffness. Under 0.10% strain, the 0.25 mol% Si doped system achieves polarization nearing 1C m^−2^, strong out of plane piezoelectricity, and reduced leakage—properties ideal for flexible electronics and energy storage devices. In contrast, 0.50 mol% Si under strain shows excessive polarization and higher leakage due to larger lattice distortion. This work provides the first demonstration that Si-doping synergistically combined with strain-engineering can unlock high-κ dielectric behavior and enhanced polarization in Cs_3_Bi_2_I_9_, establishing a new, lead-free perovskite platform for next-generation MIM capacitors.

## Abstract 2

This study examines the energy storage potential of strain engineered Si doped Cs_3_Bi_2_I_9_ perovskites using density functional theory.

## Graphical abstract

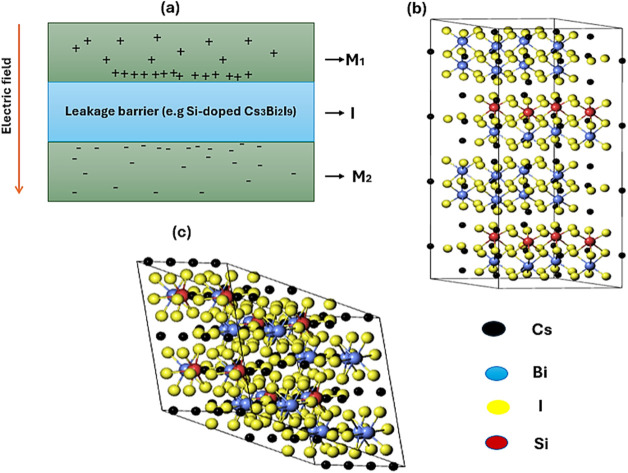

## 1 Introduction

In the global community, energy conservation and energy harvesting are crucial for many types of power sources.^1^ Batteries and capacitors are the commonly known sources of power globally, which provide a solution for the imbalance between limited energy transmission and renewable energy generation.^2^ In most cases, batteries and other metal-insulator-metal (MIM) capacitors significantly suffer from problems of low power density and efficiency due to the nature of the constituent materials.^3^ Despite this, MIM capacitors are widely adopted for various applications such as mixed integrated circuits (ICs), radio frequency (RF) circuits and sensor application devices.^4^ According to the various literature, the most challenging aspect of MIM capacitor technology is the choice of an efficient material to supress leakage with high precision.^5,6^ To contain this challenge, it is necessary to produce dielectric materials with a high polarization response under an electric field, otherwise known as field induced polarization switching.^7^ Based on high performance and durability, silicon-based materials are the most used dielectrics in MIM capacitors, because they offer high dielectric constant, excellent thermal and chemical stability, low leakage, and doping flexibility.^8,9^ Regarding this, several silicon-based oxides have been reported. For example, Soon-Jin fabricated a silicon nitride-based MIM capacitor for InGaP/GaAs HBT applications, achieving a capacitance density of 600 pF mm^−2^ with breakdown electric fields of 3.0–7.3 MV cm^−1^.^10^ Another study conducted by Federico *et al.* revealed that SiO_2_ MIM capacitors performed better in amorphous states.^11^ According to Mahata *et al.*, the performance of TaYO_*x*_ films (deposited on Au/SiO_2_) MIM capacitors depends on dielectric thickness and dielectric breakdown voltage.^12^

Despite their performance, normal silicon-based leakage barriers still suffer from polarization and charge distribution problems.^13^ They also tend to reduce performance under mechanical stress.^14^ Recently, reviews by various researchers have foreseen silicon-based perovskites as future dielectric materials, which can serve as efficient leakage barriers in MIM capacitors.^15,16^ This is because of their much higher dielectric constant than conventional silicon-based dielectrics.^17^ Unlike conventional silicon-based dielectrics, perovskite-based dielectrics are multifunctional (dielectric + semiconducting + optoelectronic) and highly compatible with emerging technologies. For example, silicon-based perovskites are being explored for flexible electronics, neuromorphic computing, and quantum devices. In this work, we investigated energy storage properties of Si-based Cs_3_Bi_2_I_9_ perovskite for efficient use as MIM capacitor. Cs_3_Bi_2_I_9_ was chosen as the model of this study due to several reasons. It is lead-free, efficient for addressing toxicity concerns unlike other materials such as MAPbI_3_.^18^ It is also a zero-dimensional layered material with isolated cluster of (Bi_2_I_9_)^3−^, with high stability than 3D perovskites.^19^ Cs_3_Bi_2_I_9_ can also accept covalent elements to break symmetry easily and enhance polarization. Various reports also revealed that Cs_3_Bi_2_I_9_ is highly stable under heat and humidity, applicable for real-world energy storage devices.^20^

## 2 Research method

All optimizations and calculations have been performed within the scope of density functional theory (DFT) implemented in quantum ESPRESSO and thermo_pw interface.^21^ The pristine system of Cs_3_Bi_2_I_9_ perovskite was generated from the materials project data base, with lattice parameters and coordinates based on the experimental alignments.^22^ Consequently, the structure was fully relaxed, until the minimum energy on each atoms becomes 0.02 eV per atom. To obtain a balance between computational cost and accuracy, convergence criterion was followed by performing convergence tests (ecut and *K*_points).^23^ For the ecut convergence, a converged value of 60 Ry was set up after running 20 Ry, 30 Ry, 40 Ry, 50 Ry, 60 Ry, 70 Ry and 80 Ry. Similarly, *K*_point converged value was set up at 9 × 9 × 9, after performing several *K*_point tests. Electronic and ionic interactions were determined using the generalized gradient approximation (GGA), in terms of Perdew–Burke–Emzerhop (PBE) norm-conserving pseudopotentials.^24^ Because GGA underestimates band gap values, we employed DFT+U, in terms of Hubbard parameters, for the atoms with d’ orbitals. Although, no literature supports adding U values of Cs and I atoms. Activating DFT+U in quantum espresso assists in obtaining relatively precise band gap values with U correction. Moreover, the U value of Si is chosen as zero because Silicon (Si) has no localized d or f electrons. It cannot be applied to s/p orbitals because it is main to correct strong on-site Coulomb interactions in localized d or f manifolds. Justification for U values in relation to the current work is shown in Table 1. To further screen the accurate band gap of the systems, hybrid HSE06 functional has also been used, and the results are compared with the GGA obtained ones.

**Table 1:** Electronic configurations and justification for DFT+U approach

Element	Electronic configuration/orbital	Recommended U (eV)	Justification
Cs	1s^2^ 2s^2^ 2p^6^ 3s^2^ 3p^6^ 4s^2^ 3d^10^ 4p^6^ 5s^2^ 4d^10^ 5p^6^ 6s^1^	0.00	Cs d-states lie deeply within the core; previous reports on Cs-based halide perovskites never applies U to Cs
Bi	1s^2^ 2s^2^ 2p^6^ 3s^2^ 3p^6^ 3d^10^ 4s^2^ 4p^6^ 4d^10^ 5s^2^ 5p^6^ 4f^14^ 5d^10^ 6s^2^ 6p^3^	0.00	Band edges are dominated by Bi 6p-statesd and are weakly correlated; most works use no U, some apply small U (<2 eV) to correct band gaps
I	1s^2^ 2s^2^ 2p^6^ 3s^2^ 3p^6^ 3d^10^ 4s^2^ 4p^6^ 4d^10^ 5s^2^ 5p^5^	0.00	Halide p states are uncorrelated; no literature supports U on I in halide perovskites

To fabricate Si-based MIM leakage barrier, Si atoms were introduced into Cs_3_Bi_2_I_9_ lattice in concentrations of 0.25 and 0.50 mol% respectively and the systems underwent relaxation again. To accurately assess the thermodynamic stability of Si substitution in Cs_3_Bi_2_I_9_, the formation energy was computed using the standard defect-formation formalism referenced to elemental chemical potentials. For Si substituting Bi, the formation energy is given by^25^

1 *E*_f_ = *E*_tot_(Si) − *E*_tot_(prisitne) − *µ*_Si_ − *µ*_Bi_

where *E*_tot_(Si) is the total energy of the relaxed Si-doped Cs_3_Bi_2_I_9_ supercell, *E*_tot_(pristine) is the total energy of the undoped supercell, and *µ*_Si_ and *µ*_Bi_ are the chemical potentials of the respective atomic species. The elemental reference states are taken as: bcc-Cs(s), rhombohedral-Bi(s), I_2_(g) for iodine, and diamond-Si(s). To ensure thermodynamic stability, the chemical potentials are constrained by the Cs_3_Bi_2_I_9_ stability condition:

2 3*µ*_Cs_ + 2*µ*_Bi_ + 9*µ*_I_ = Δ*H*_f_(Cs_3_Bi_2_I_9_)

with additional inequalities preventing decomposition into competing Cs–Bi–I secondary phases.

Electronic interactions, density of states and orbital interactions were determined from the density of state parameter^26^3
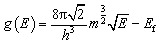
where *h* is the plank's constant. We analyze the charge polarizing parameter in terms of the born effective charges and charge density calculations. Polarization, electric field and dielectric behaviors of the systems were considered to fully understand the energy storage capacity of the MIM system under investigation.

The strained-induced charge storage properties of the systems were equally calculated by introducing 0.10% strain on the *z*-direction. Consequently, parameters such as piezoelectric coefficients, leakage and capacitance have been determined for the strain-induced systems.

## 3 Results and discussions

### 3.1 Structural parameters

**Fig. 1:**
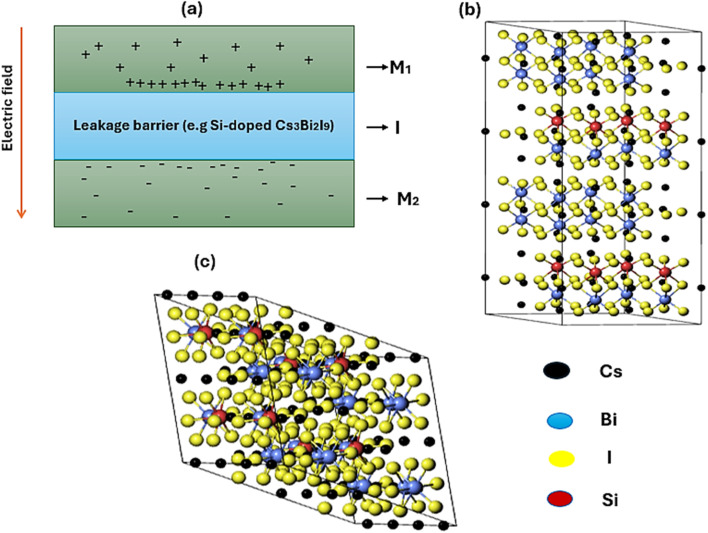
(a) Schematic illustration of the Si based Cs_3_Bi_2_I_9_ MIM capacitor, showing how electric field is set up due to voltage blockage in the leakage barrier (b) Si based Cs_3_Bi_2_I_9_ crystal in *XY* viewed in *XY* plane (c) Si based Cs_3_Bi_2_I_9_ crystal in *XYZ* viewed in *XY* plane.

Fig. 1(a) depicts a schematic layout of a typical MIM capacitor commonly used in microelectronics and energy storage.^27^ The top layer labeled *M*_1_ acts as the positive terminal of the capacitor, which accumulates positive charges due to applied external voltage. The insulating layer (I) is the region which prevents leakage current by preventing direct charge flow. Otherwise, this dielectric layer stores excess energy by setting up electric field flow. On the other hand, *M*_2_ acts as the second or negative terminal of the capacitor, which accumulates negative charges due to external voltage. As shown by the arrow direction, electric field is established across the insulator when external voltage is applied. The structural geometry of the Si based MIM capacitors are shown in Fig. 1(b and c), indicating *XY* and *XYZ* planes.

The original lattice of the pure Cs_3_Bi_2_I_9_ perovskite was a hexagonal crystalline perovskite obtained from materials project data base. Cs_3_Bi_2_I_3_ adopts a hexagonal *P*6_3_/*mmc* structure containing two distinct Cs^+^ sites. In the first site, Cs^+^ coordinates with twelve I^−^ ions in a CsI_12_ cuboctahedron that connects through corners and faces to neighboring CsI_12_ units and BiI_6_ octahedra, with octahedral tilt angles of about 19° and Cs–I distances of 4.22–4.49 Å. The second Cs^+^ site also forms a CsI_12_ cuboctahedron but with six shorter (4.27 Å) and six longer (4.37 Å) Cs–I bonds. Bi^3+^ forms BiI_6_ octahedra exhibiting three short (2.96 Å) and three long (3.23 Å) Bi–I bonds. Two inequivalent I^−^ sites show 1- and 2-coordinate geometries. For the purposes of this work, a 2 × 2 × 2 supercell containing 224 atoms was optimized based on lattice parameters *a* = 8.54 and *c* = 2.16 Å respectively.^28^ Before relaxing, the original volume of the super cell model was 2732.54 Å^3^. Later, the supercell was appropriately relaxed until the minimum energy per atom becomes 0.02 eV per atom. After relaxation, Pristine Cs_3_Bi_2_I_9_ retained the centrosymmetric space group *P*6_3_/*mm*, while 0.25 mol% Si-doped Cs_3_Bi_2_I_9_ transformed to non-centrosymmetric space group *P*6_3_*mc*. The observed transformation reveals loss of inversion symmetry due to asymmetric Si substitution within the Bi_2_I_9_ cluster.

Since Si^4+^ and Bi^3+^ are hetero-valent, doping with Si presented a relatively complex structure because Si^4+^ has smaller ionic radius than Bi^3+^ and Cs.^29,30^ So, energy did not converge directly when Si substitutes Bi atoms. On this basis, the Si–Ti bonding process presented a significant lattice distortion with compensation by creating one positive charge (hole) according to

4 Si_Bi_^4+^ → Bi_Bi_^3+^ + *h*

The lattice compensates this charge primarily through formation of iodine vacancy, which are the most common and lowest-energy compensating defects in halide perovskites. Therefore, the electronic and other properties obtained for Si-doped Cs_3_Bi_2_I_9_ perovskites were based on iodine vacancy. The effects of 0.25 and 0.50 mol% Si atoms produced some decrease in the volume of the unit cell to 2645.78 and 2607.81 Å^3^ respectively, because the ionic radius of Si atom is comparatively small to ionic radius of Bi atom. Bi–I bond length also elongates on the site of Si.^31^

To determine thermodynamic stability of the current systems, we considered formation energy parameter as shown in Table 2. Notably, Si substitution is energetically favourable within the full chemical-potential stability range of Cs_3_Bi_2_I_9_. Under both I-rich and Bi-rich growth limits, the doped structures exhibit negative formation energies, confirming intrinsic thermodynamic stability. The 0.25 mol% Si configuration remains the most stable, consistent with observed structural relaxation and reduced lattice volume.^32^

**Table 2:** Structural parameters of the MIM systems under investigation

MIM systems	*E*_f_ (eV)	*a*; *c* (Å)	Volume (Å^3^)
Cs_3_Bi_2_I_9_	−0.96	8.54; 2.16	2673.44
0.25 mol% Si@Cs_3_Bi_2_I_9_	−1.15	8.52; 2.15	2645.78
0.50 mol% Si@Cs_3_Bi_2_I_9_	−1.20	8.49; 2.09	2607.81

Additionally, the trend of the formation energy becomes more negative as Si concentration increases. Therefore, doping with Si leads to some thermodynamic stability especially in the 0.25 mol% variant. Slight decrease in lattice constants indicates lattice contraction due to smaller ionic radius of Si compared to Bi. Ultimately, the improved stability due to Si doping indicates a potentially good structural integrity under electric fields. On the other hand, reduced volume tightens the crystal lattice, which may lead to higher dielectric constants. On the overall notice, the progressive improvement from 0.25 to 0.50 mol% Si shows that controlled doping can tune material properties for optimal capacitor performance.

### 3.2 Initial electronic properties

**Fig. 2:**
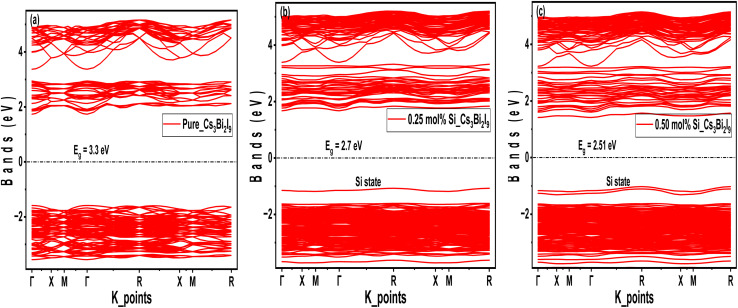
GGA band structure diagrams of the Cs_3_Bi_2_I_9_ MIM capacitor materials, each demonstrating energy bands at various energy levels (a) pure (b) 0.25 mol% Si (c) 0.50 mol% Si.

Analyzing electronic properties of systems is highly significant in reporting their potential in MIM capacitor applications. For example, studying band gap parameters helps understand the materials metallicity, resistivity, work function and band alignment mechanisms necessary for efficient charge storage.^33^ As presented in Fig. 2(a–c), the band structure diagrams for pure and Si-based Cs_3_Bi_2_I_9_ variants identify important trends directly connected to properties of MIM capacitors, especially work function and band alignment. In the pure system, a wide band gap of 3.32 eV (3.47 eV with HSE06) suggests low intrinsic conductivity coupled with high voltage breakdown. This may lead to limitation in charge of injection efficiency. Significant band gap narrowing (2.70 eV GGA) and (2.82 with HSE06) were observed when 0.25 mol% Si atoms were introduced, which shows better charge carrier facilitation with improved polarization response. The corresponding band gaps for the 0.5 mol% Si-doped variants are (2.51 eV) and (2.59 eV) with GGA and HSE06 respectively. We also observed stronger electronic interaction due to additional Si atoms, which can influence materials dielectric behavior. Based on the information obtained in the band structure diagrams, presence of Si dopants in Cs_3_Bi_2_I_9_ strategically regulates the band structure to optimize work function and band alignment. Collectively, this can tune to the material potential for high-performance MIM capacitors. Notably, 0.25 mol% Si-doped variant offers the best balance between electronic activity and dielectric stability.

**Fig. 3:**
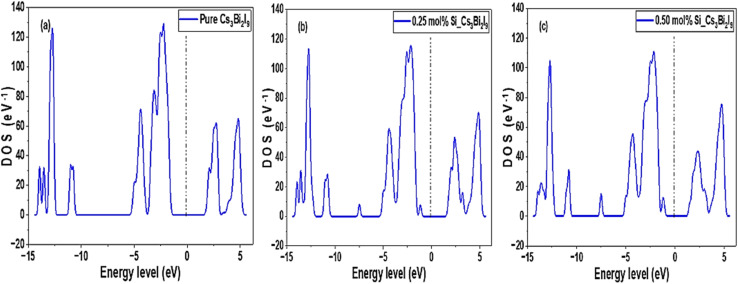
DOS pattern of the Cs_3_Bi_2_I_9_ MIM capacitor materials, each demonstrating the level of energy states occupations at various energy levels (a) pure (b) 0.25 mol% Si (c) 0.50 mol% Si.

The DOS diagrams presented in Fig. 3(a–c) revealed some number of electronic states available at various energy levels. As observed in Fig. 3(a), a clear indication of separation between valence band and conduction band emerged and relatively wide, indicating wide band gap in pure Cs_3_Bi_2_I_9_. In Fig. 3(b) DOS peaks shifted slightly towards Fermi level, indicating band gap narrowing. Moreover, peaks near Fermi level shifted more and broader due to increase in Si concentration, as shown in Fig. 3(c). The mechanism of activities near Fermi level in the Si based Cs_3_Bi_2_I_9_ MIM capacitors are subsequently explained using partial density of states (PDOS) analysis.

**Fig. 4:**
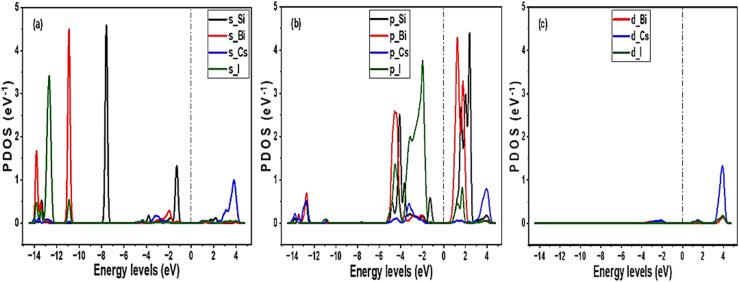
PDOS pattern of the Cs_3_Bi_2_I_9_ MIM capacitor materials, each demonstrating various orbital contributions at various energy levels (a) pure (b) 0.25 mol% Si (c) 0.50 mol% Si.

The PDOS pattern shown in Fig. 4(a–c) demonstrates various orbital activities in engineering electronic properties of the Si-based Cs_3_Bi_2_I_9_ MIM capacitors. In Fig. 4(a), core energy levels (−12–−14 eV) are dominated by 5s (Cs) and 6s (Bi) orbitals. Therefore, core bands are primarily dominated by Cs 5s, Bi 6s, and I 5s orbitals, which can enhance stability and leakage suppression. As shown in Fig. 4(b), there is significant activity of p′ orbitals of Si and Bi atoms near Fermi level. Also, Si p′ state creates new state near CBM, which effectively narrows the band gap. Additionally, p′ orbitals of Bi and I contributed to the VBM, collectively forming the lower edge band gap. Notably in Fig. 4(c), d′ orbitals demonstrated minor contributions in the mid-gap state, hence considered less influential in the band gap formation.

#### 3.2.1 Initial static dielectric constants

**Fig. 5:**
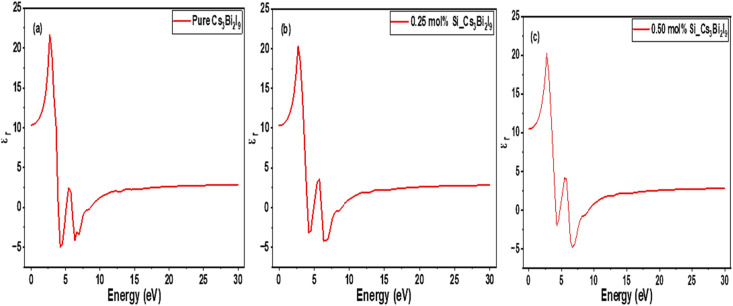
Initial static dielectric constant spectra of Cs_3_Bi_2_I_9_ MIM capacitor materials (a) pure (b) 0.25 mol% Si (c) 0.50 mol% Si.

Dielectric behavior of MIM systems relates their response to external electric fields across various energy levels.^34^ In the current work, detailed discussions on the dielectric behaviors of Cs_3_Bi_2_I_9_ MIM capacitors are done based on results referred in Fig. 5(a–c). In MIM capacitor, high and stable dielectric constant indicates improved charge storage and efficiency.^35^ As shown in Fig. 5(a), pure Cs_3_Bi_2_I_9_ revealed moderate response in the range 4–6 eV, suitable as a baseline insulator. However, its efficiency can be low due to strong polarization in the UV range. We observed enhanced dielectric response with 0.25 mol% system as shown in Fig. 5(b). Higher peak and broad energy range (2.5–5.4 eV) indicates increased polarization because of defect induced dipoles, localized lattice distortions and charge redistribution mechanism.^36^ Notably, Si doping level (0.25 mol%) significantly improves energy storage efficiency with high chance of reduced leakage, relative to MIM capacitor applications. 0.5 mol% doping level (Fig. 5(c)) presented significantly modified profile with very little shift in the dielectric peak. The little shift in the dielectric peak suggests defect clustering with enhanced localized states. The overall mechanisms of dielectric response observed are due to electronic polarization (see Table 5) which is dominant at high frequencies; leading to band gap narrowing, which can increase carrier mobility and charge compensation mechanisms between Si^4+^ and Bi^3+^ ions. Based on our findings, 0.25 mol% Si-doped Cs_3_Bi_2_I_9_ offers better balance between leakage control and dielectric enhancement. Additionally, 0.5 mol% system may need careful defect control to prevent excessive leakage.

#### 3.2.2 Born effective charge (BEC) distribution

**Fig. 6:**
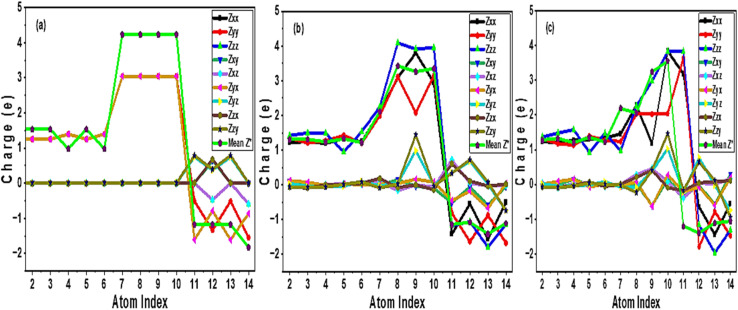
Variation of BEC with atom indices of Cs_3_Bi_2_I_9_ MIM capacitor materials (a) pure (b) 0.25 mol% Si (c) 0.50 mol% Si.

BEC measures the change in crystal polarization when atom is displaced.^37^ We analyse BEC distribution based on the calculated dielectric tensors, which reflects the dynamical activities of the electronic systems. Fig. 6(a–c) present BEC graph for pure and Si based Cs_3_Bi_2_I_9_ MIM capacitor systems. The atomic indices for pure Cs_3_Bi_2_I_9_ are: 2 = Cs; 3 = Cs; 4 = Cs; 5 = Cs; 6 = Cs; 7 = Si; 8 = Bi; 9 = Bi; 10 = Bi; 11 = I; 12 = I; 13 = I; and 14 = I; The corresponding atomic indices for 0.25 mol% and 0.50 mol% Si doped systems are obtained by replacing Bi index number (7) and (7 & 8) with Si atoms respectively. Fig. 6(a) depicts BEC tensor components of pure Cs_3_Bi_2_I_9_*vs.* atom indices in the range 2–14. Each line in the graph represents tensor components *Z*_*xx*_, *Z*_*yy*_, *Z*_*zz*_, *Z*_*xy*_, *Z*_*yx*_, *Z*_*xz*_, *Z*_*yz*_ and their mean values. Notably, all diagonal components presented directional polarization due to moderate values across indices 2–10. On the other hand, off diagonal components (*Z*_*xy*_, *Z*_*yx*_, *Z*_*xz*_, *Z*_*yz*_) demonstrate low anisotropic polarization. Primary polarizations are shown by indices 7–9 (Bi atoms), while indices 11–14 (I atoms) show negative charge values, efficient for charge compensation. Relatively constant mean charge values indicate low dynamic response in the pure Cs_3_Bi_2_I_9_ perovskite. Generally, relatively stable BECs in pure Cs_3_Bi_2_I_9_ reveals lower response to external fields.

In Fig. 6(b), Si atom (index 7) demonstrates a notable stronger diagonal component (*Z*_*xx*_, *Z*_*yy*_ and *Z*_*zz*_) indicating a higher local polarization. By introducing local polarization, there will be symmetry breaking and strong dipole formation, crucial for efficient dielectric performance. Indices 1–8 (Bi atoms) presented relatively high BEC values specifically in *Z*_*zz*_ and *Z*_*xx*_. Indices 2–6 (Cs atoms) show medium and consistent BEC values efficient for supporting structural symmetry rather than taking role in active polarization. Charge compensation mechanisms are observed with indices 11–14 (I atoms) because of negative values of BEC, efficient for electrostatic stability and suppressing dielectric loss. The obtained high mean Z* attributed to Si and Bi sites suggests strong dielectric activity, necessary for high-performance capacitors. Ultimately, adding 0.25 mol% Si in Cs_3_Bi_2_I_9_ creates localized polarization centres and improves dielectric anisotropy, highly suitable for MIM capacitor materials where efficiency, miniaturization, and fast switching are critical. In the 0.50 mol% doped system, dual polarization centre emerged because of presence of more Si atoms. Therefore, there is highly distributed polarization coupled with stronger mean values and diagonal components. The dual-centre behaviour increases dielectric symmetry, which is significant for energy storage and minimizing local field concentration. As shown in Table 3, higher mean values for Si-doped systems confirmed strong response to polarization, while lower and negative mean value obtained from pure system suggest reduced net polarization. Therefore, the obtained BEC properties of pure Cs_3_Bi_2_I_9_ inclined its application for non-tuneable high capacitance applications, while BEC properties attributed to Si-variants of Cs_3_Bi_2_I_9_ suggests their application as tuneable capacitors, memory devices, or nonlinear dielectric applications.

**Table 3:** BEC values of some high-level tensor components of the MIN capacitors under investigation

Systems	*Z*_*xx*_ (e)	*Z*_*yy*_ (e)	*Z*_*zz*_ (e)	*Z*_*xy*_ (e)	*Z*_*yz*_ (e)	Mean value (e)
Pure Cs_3_Bi_2_I_9_	0.04957	0.04934	0.02819	−0.00000	−0.00000	−0.01680
0.25 mol% Si@Cs_3_Bi_2_I_9_	−0.00562	−0.03521	−0.00958	0.02371	0.02411	1.34494
0.50 mol% Si@Cs_3_Bi_2_I_9_	−0.00254	−0.03699	−0.00667	0.02645	0.00043	1.34577

#### 3.2.3 Charge density distribution

**Fig. 7:**
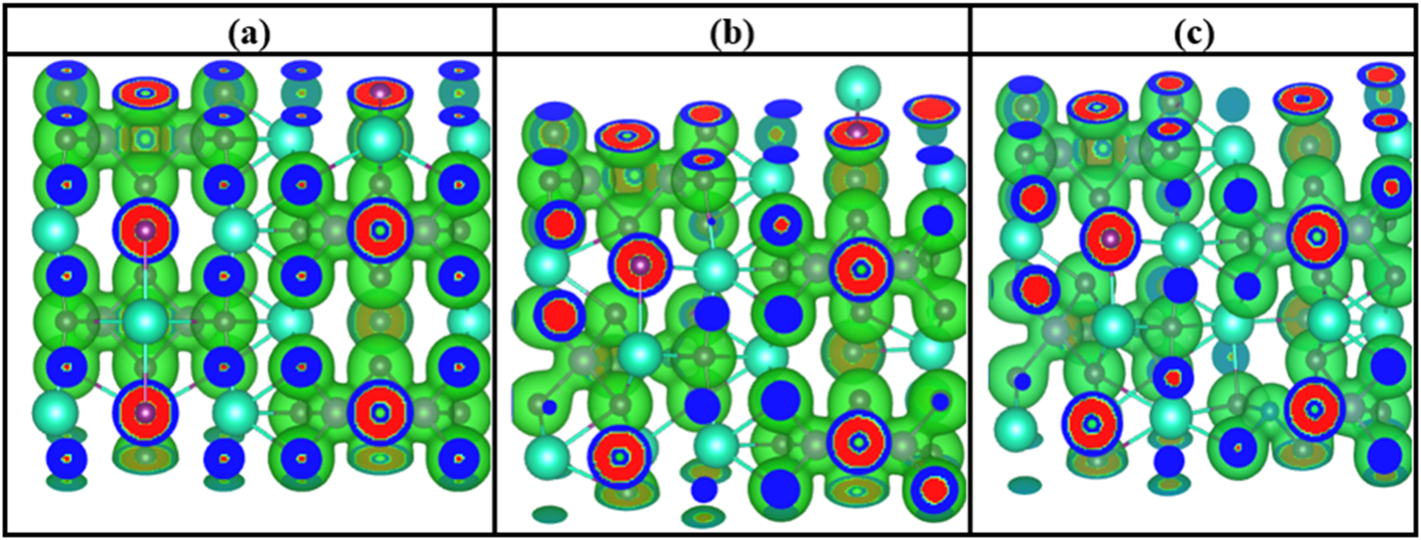
Charge density distribtion of Cs_3_Bi_2_I_9_ MIM capacitor materials (a) pure (b) 0.25 mol% Si (c) 0.50 mol% Si.

The concept of charge density distribution enables better understanding of how electrons are distributed in Cs_3_Bi_2_I_9_ and its Si doped variants.^38^ It also highlights bonding characteristics, electronic interactions and centers of polarization. For better understanding of dielectric behaviors of these systems, we used charge density approach to identify regions of high and low electron localization and the significance of Si dopants. In the charge density diagram provided in Fig. 7(a), blue regions identify sparse electrons in the interstitial nonbonding regions. In the doped systems as shown in Fig. 7(b and c), it indicates where charges are pulled away because of polarization influence near Si site. Areas of high electron density are indicated by red zones. They typically represent active electronic environments and polarization centers.

Notably, pure Cs_3_Bi_2_I_9_ perovskite reveals relatively uniform charge density distribution, showing high localized regions around Bi and I atoms. Due to this, the charge density distribution for this system suggests stable electronic environment with well-defined bonding. There is significant charge enhancement near Si site in the 0.25 mol% system due to presence of single red region, which is efficient for improving dielectric properties. We also observed slight asymmetry due to presence of Si atoms, confirming local distortion and incrased dipole moments.^39^ By increasing Si concetration to 0.50 mol%, charge density becomes more intense around Si atoms. For example, two distinct red regions appeared near both Si atoms, signifying improved and spread charge localization. The system also shows greater anisotropy leading to high field response.

### 3.3 Unstrained mechanical properties

**Fig. 8:**
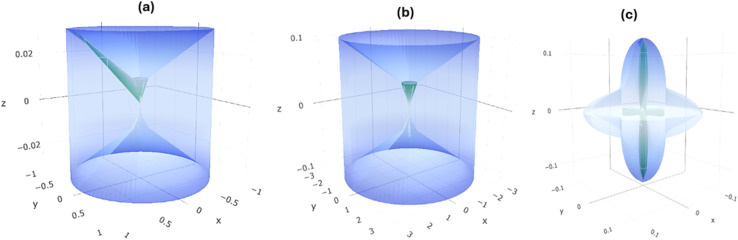
Spacial dependence of shear moduli of Cs_3_Bi_2_I_9_ MIM capacitor materials (a) pure (b) 0.25 mol% Si (c) 0.50 mol% Si.

Describing mechanical parameters such as shear moduli and Poisson's ratio can explain mechanical stability and how dielectric materials respond to voltage induced mechanical stress.^40^ Based on the related previous data, optimum balance between electrical and mechanical properties leads to performance consistency.^41^ A 3D visualization of the shear moduli for Cs_3_Bi_2_I_9_ MIM capacitors under investigation is presented in Fig. 8(a–c), according to the relation^42^5
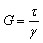
where *τ* is the shear stress and *γ* is the shear strain. A cylindrical shape with green surface intersecting the cylinder indicates unifiom shear modulus across directions. A slight asymmety was observed in Fig. 8(b), specifically along *z*-direction. The green surface is titled more than in Fig. 8(a), showing the emergence of anisotropy. Hence Si atoms intoroduced directional dependence stiffness bacause of lattice distortions. Ellipsoidal pattern shows stronger anisotropy in Fig. 8(c), which is also confirmed by intersecting planes along *x*, *y* and *z*-axies. Acoording to Liang *et al.*, anisotopy is significant for nonlinear dielectric materials but requires good management under high electric fields.^43^ The tabulated calculated values of the directional dependence mechanical properties of the current systems are shwon in Table 4. Notably, highest values of stiffnesss, shear moduli and Poisson's ratio values are typical for pure Cs_3_Bi_2_I_9_. Although good for structural stability, low stiffness is necessary for flexible MIM capacitors. Ehanced flexibility was observed in 0.25 mol% Si-based system, due to reduced values of stiffness and shear moduli. The 0.50 mol% MIM system is extremely mechanically soft. With careful mechanical design, it can be useful for strain sensitive electronics.

**Table 4:** Tensile properties of the Cs_3_Bi_2_I_9_ MIM capacitors

MIM systems	*Y* (GPa)		*G* (GPa)		(*P*)	
	Min	Max	Min	Max	Min	Max
Pure Cs_3_Bi_2_I_9_	0.003999	183.65	0.001	66.4	0.0000038	0.99998
0.25 mol% Si@Cs_3_Bi_2_I_9_	0.014239	137.27	0.00356	50.195	0.0000037	0.99992
0.50 mol% Si@Cs_3_Bi_2_I_9_	0.011259	0.19804	0.00291	0.12518	−1.6218	2.0873

**Fig. 9:**
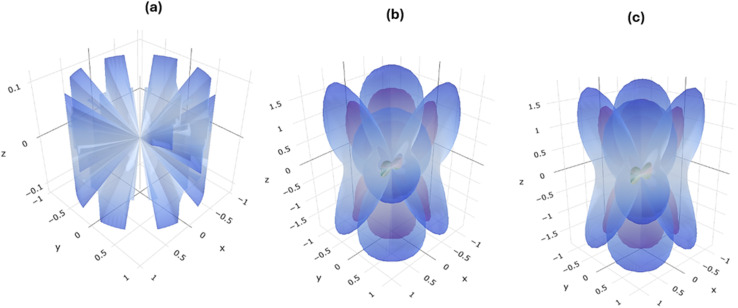
Spacial dependence of Poisson's ratio of Cs_3_Bi_2_I_9_ MIM capacitor materials (a) pure (b) 0.25 mol% Si (c) 0.50 mol% Si.

In Fig. 9(a), highly symmetric pattern of Poisson's ratio confirms isotropic response. Therefore, pure Cs_3_Bi_2_I_9_ responds uniformly under mechanical stress. However, anisotropic response was confirmed as shown in Fig. 9(b), due to apperance of irregular overlapping lobes caused by 0.25 mol% Si atoms. This observed behaviours can influence stress distribution in the dielectric layer, potentially influencing dielectric breakdown or fatigue resistance. Accroding to Fig. 9(c), the 0.50 mol% Si-based MIM system shows a moderately symmetric property, suggesting a patically restored symmetery. The behavoiur attributed to this system ehnaced its mechanical compliance necessary to maintain electrical performance.

### 3.4 Initial dielectric constant, electric field and polarization tensors

In the concept of MIM capacitor materials, we provided highlights of how Si atoms influences dielectric properties of Cs_3_Bi_2_I_9_ perovskite. We determine the capacitance of the MIM sysrems under investigation from the expression^44^6
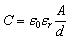
where *A* and *d* respresent area and thickness of the parallel metal electrodes in the MIM systems. *ε*_*r*_ is the relative permittivity obtained form the dielectric tensor data from Table 4. We also determined electric field strength of the corresponding MIM systems by assuming electric dipslacement field *D* = (1, 1, 1) × 10^−12^ Cm^−2^, according to the formular^45^7
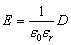


Based on the calcualted values in Table 5, capacitance (*C*) is directly proportional to dielectric constants. Notably, there is high increase in *ε* values due to introduction of Si impurities, leading to higher capacitance. On the other hand, *E* was inversely proportional to *ε* values in all anisotropic directions. As *ε* increase, there is corresponding decrease in the *E* values required to maintain same charge displacement. Based on previous reports, the observed phenomena is necessary for low leakage risks and better riliability.^46^ Pure Cs_3_Bi_2_I_9_ demonstrates the lowest *C* and *E* values, whereas 0.50 mol% Si based variant reveals highest *C* and relatively reduced *E* values compared to 0.25 mol% Si based variant, which is an indication of dielectric screening, ideal for high performance MIM capacitors.^47^ To determine polarization effects, we adopt expression of polarization to electric field as presented in eqn (8)8
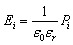
where *ε*_0_ is the vacuum permittivity space (8.854 × 10^−12^ F m^−1^) and *ε*_*r*_ is the relative permittivity. Calculation was done using the modern Berry-phase formalism, which correctly yields zero polarization for centrosymmetric pristine Cs_3_Bi_2_I_9_ and finite polarization only for doped structures with verified non-centrosymmetric symmetry. To determine whether Si doping induces global symmetry breaking necessary for ferroelectric or piezoelectric behavior, we performed full point-group and space-group analysis using the spglib implementation in Thermo_pw. The emergence of finite macroscopic polarization and non-zero piezoelectric tensors in the Si-doped systems is therefore fully consistent with the observed global symmetry breaking. Data from the microscopic dipole distribution (Table 6) due to Si-doping in Cs_3_Bi_2_I_9_ reveals a clear alignment with the enhanced dielectric response previously observed. In the pristine system, there is negligible out-of-plane (*z*-direction) contribution, consistent with the centrosymmetric nature of the *P*6_3_/*mmc* phase, prohibiting spontaneous polarization. When 0.25 mol% Si were introduced, the *z*-component increases from near zero to approximately 7.46 e·Å, corresponding to 0.0447C m^−2^. This increase confirms that substitutional Si breaks the local inversion symmetry within the Bi_2_I_9_ polyhedral units by inducing asymmetric charge redistribution and selective bond shortening. This collectively promote off-cantering of both cations and anions. Moreover, these microscopic distortions align with the enhanced Born effective charges and charge-density asymmetries reported earlier in the manuscript.

**Table 5:** Dielectric tensors and energy storage deriven paramters of the cureent Cs_3_Bi_2_I_9_ MIM capacitors

Systems	*ε*_*xx*_	*ε*_*yy*_	*ε*_*zz*_	*C*_*xx*_; *C*_*yy*_; *C*_*zz*_ (µF cm^−2^)	*E*_*xx*_; *E*_*yy*_; *E*_*zz*_ (Vm^−1^)
Pure Cs_3_Bi_2_I_9_	4.02	4.02	4.23	3.75; 3.75; 3.55	3.69; 3.69 × 10^−7^; 1.08 × 10^−9^
0.25 mol% Si@Cs_3_Bi_2_I_9_	5.09	5.09	4.66	4.51; 4.51; 4.13	2.22 × 10^10^; 2.22 × 10^10^; 2.42 × 10^10^
0.50 mol% Si@Cs_3_Bi_2_I_9_	5.22	5.22	4.79	4.62; 4.62; 4.24	2.16 × 10^10^; 2.16 × 10^10^; 2.36 × 10^10^

**Table 6:** Comparison of dipole vectors and polarization for pristine and Si-doped Cs_3_Bi_2_I_9_, showing how substitutional Si introduces and amplifies local polar distortions that drive enhanced dielectric and polarization responses

MIM systems	*µ* (e.Å)	*µ* (Debye)	∼*P* (Cm^−2^)
Pure Cs_3_Bi_2_I_9_	(−8.4283, −14.5982, ∼0)	(−40.48, −70.12, ∼0)	(−0.05046, −0.08740, ∼1.7 × 10^−9^)
0.25 mol% Si@Cs_3_Bi_2_I_9_	(−4.2141, −12.1652, 7.4626)	(−20.24, −58.43, 35.84)	(−0.02523, −0.07283, 0.04468)
0.50 mol% Si@Cs_3_Bi_2_I_9_	(−4.2141, −7.2991, 21.7287)	(−20.24, −35.06, 104.37)	(−0.02523, −0.04370, 0.13009)

The effect becomes significantly amplified with 0.5 mol% Si. The *z*-dipole rises to 21.73 e Å, more than the magnitude observed in the 0.25 mol%-Si case, producing polarization of approximately 0.13C m^−2^. This pronounced increase highlights the cooperative nature of Si-induced polar distortions: multiple Si sites reinforce each other through lattice-mediated long-range electrostatic interactions, driving stronger symmetry breaking across the supercell. Meanwhile, the in-plane dipole components systematically decrease, suggesting partial recovery of lateral symmetry even as vertical asymmetry intensifies. Collectively, these trends demonstrate that Si substitution introduces robust polar centres, offering a compelling microscopic explanation for the experimentally relevant enhancement of dielectric, polarization, and piezoelectric properties in Si-doped Cs_3_Bi_2_I_9_.

**Fig. 10:**
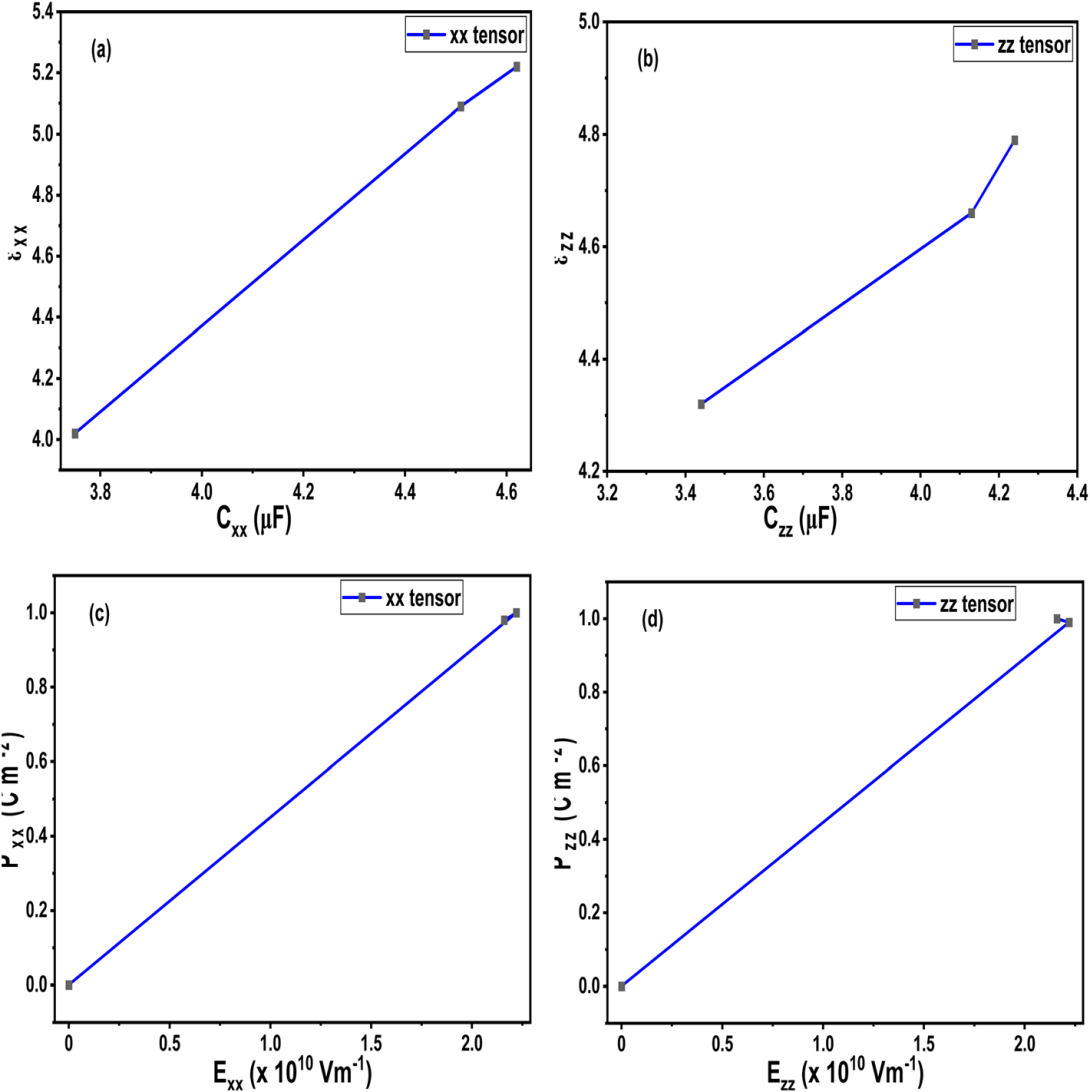
Variation of capacitance with dielectric constants (a) *x*-direction (b) *y*-direction. Variation of electric field with polarization (c) *x*-direction (d) *y*-direction. The plotted graphs are based on the calculated values of Tables 4 and 5.

Fig. 10(a–d) present a graphical visualization of the components of dielectric tensors, capacitance and electric fields. Fig. 10(a) indicates the existence of linear relationship capacitance and dielectric tensor in *x*-direction, indicating that more energy per unit voltage is stored according to theoretical equation9
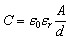


*z*-tensor component also shows increasing trend with non-linear deviation at higher dielectric values, indicating saturation in *z*-direction due to structural distortion by Si atoms. In Fig. 10(d), linear variation between polarization and electric field suggests field-induced polarization, aligning with field strength. It shall be noted that properties in *y*-direction are not reported because *x*- and *y*-directions are isotropic (see Table 5)

### 3.5 0.10% strained energy storage analysis

**Fig. 11:**
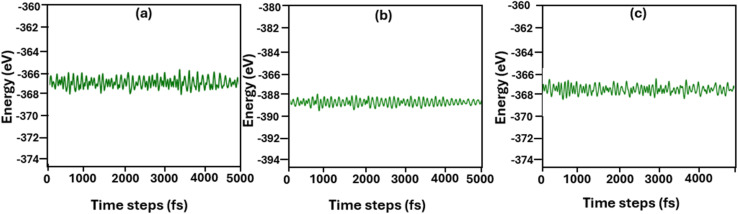
Energy variations for the Cs_3_Bi_2_I_9_ perovskite and its Si-doped variants under 300 K. (a) Pure (b) 0.25 mol% Si-doped (c) 0.50 mol% Si-doped.

The concept of strained energy storage properties of the current systems under study cannot be overlooked. Applying external strain to energy storage materials modifies orbital overlap and charge distribution, which boosts their dielectric response.^48^ Strained electronic devices also exhibit better piezoelectric properties due to increased polarization.^49^ However, applying strain requires very careful procedure because excess strain can compromise the system's mechanical stability. In the current study, we applied 0.10% strain on the *z*-direction of the three Cs_3_Bi_2_I_9_ MIM systems and subsequently observe changes in various energy storage parameters such as band gap, charge density, polarization, electric field and piezoelectric response. The *ab initio* molecular dynamics (AIMD) results for pure and doped Cs_3_Bi_2_I_9_ systems are displayed in Fig. 11(a–c). results have been achieved by performing AIMD simulations at 300 K under the condition *x* = 2. In all three systems, the total energy exhibit only minor fluctuations throughout the simulation, indicating strong thermal stability of the structures, consistent with previous findings.^50^ Maintaining structural integrity at 300 K is particularly important, as it demonstrates that the investigated Cs_3_Bi_2_I_9_ systems can operate safely under typical capacitor operating conditions.

#### 3.5.1 Strained electronic properties

**Fig. 12:**
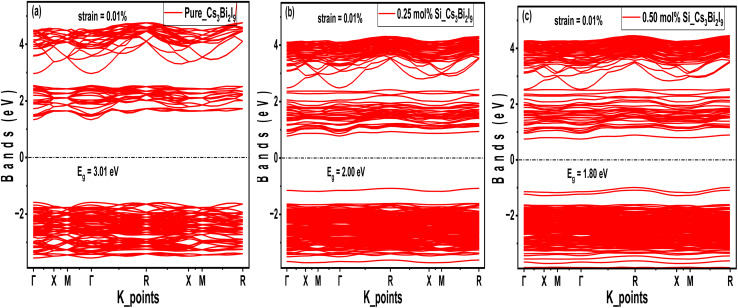
0.10% strained band structure diagrams of the Cs_3_Bi_2_I_9_ MIM capacitor materials, each demonstrating energy bands at various energy levels (a) pure (b) 0.25 mol% Si (c) 0.50 mol% Si.

To align with the concept in this research work, we take account of the changes on the electronic properties after introducing 0.10% strain along *z*-direction. According to some reports, strain induced electronic systems undergo significant change in polarization and charge storage capacity.^51,52^Fig. 12(a–c) display the obtained strained electronic band structures of the Cs_3_Bi_2_I_9_ MIM systems under investigation. Compared to initial systems (refer to Section 3.2), we observed slight narrowing and broadening in band gap due to stress-induced shift of some energy bands. For example, the initial band gap of the pure Cs_3_Bi_2_I_9_ which was 3.3 eV, is slightly narrowed to ∼3.2 eV after 0.10% strain was induced to the system. The observed band gap value has brought Cs_3_Bi_2_I_9_ to moderate insulation and low leakage range, ideal for high-performance MIM capacitors.^53^ Although its band gap decreased, the probability of excessive leakage is still low since band gap is still wide. In Fig. 12(b and c), 0.25 mol% Si based Cs_3_Bi_2_I_9_ experienced some widening of band gap, to new value of ∼2.8 eV, while some decrease to ∼2.4 eV is attributed to 0.50 mol% Si based Cs_3_Bi_2_I_9_. At low Si content, strain slightly decreases the lattice distortion and enhances orbital alignment, which in turn reduces some defects states and restore symmetry.^54^ With this, the conduction band and valence band are pushed apart, leading to increase in band gap. Since lattice is already distorted with 0.25 mol% Si, introducing more Si concentration with 0.10 strain increases hybridization between Si 3p and Bi/I p orbitals, which pulls bands closer due to high electronic disorder within mid-gap states. Based on the results implications, 0.25 mol% can provide a balanced performance with low leakage and improved stability than 0.50 mol% Si based system. More discussion on leakage can be found under Section 3.10.

**Fig. 13:**
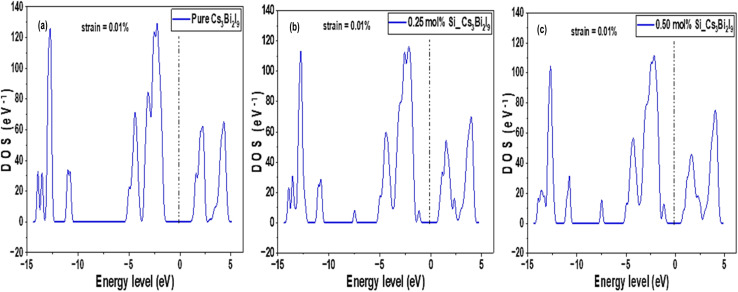
0.10% strained DOS pattern of the Cs_3_Bi_2_I_9_ MIM capacitor materials, each demonstrating the level of energy states occupations at various energy levels (a) pure (b) 0.25 mol% Si (c) 0.50 mol% Si.

**Fig. 14:**
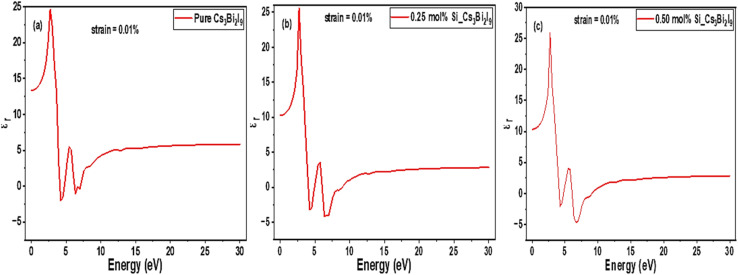
0.10% strained static dielectric constant spectra of Cs_3_Bi_2_I_9_ MIM capacitor materials (a) pure (b) 0.25 mol% Si (c) 0.50 mol% Si.

The corresponding strained DOS diagrams of these systems are shown in Fig. 13(a–c), each confirming some semiconducting properties of the strained systems. According Guancheng *et al.*, static dielectric constants of perovskites are highly sensitive to structural deformations and redistribution of electronic density.^55^ As shown in Fig. 14(a–c), all spectra exhibited relatively sharp peaks at low energy despite undergoing strain. In Fig. 14(a), we observed minimal change in dielectric spectra because Cs_3_Bi_2_I_9_ remains largely centrosymmetric. Abrupt drop in the peak to near zero suggests limited polarizability and weak dielectric response under strain.^56^ This is because of the centrosymmetric structure, which suppresses ionic displacement during strain. The peak of 0.25 mol% Si system rise to new high intensity and then becomes broader at low energy region, suggesting enhanced polarizability (see polarization). In Fig. 14(c), peak maintains highest value with greater fluctuations due to high anisotropy, which may also indicate some instability due to effects of excess Si atoms and strain.

**Fig. 15:**
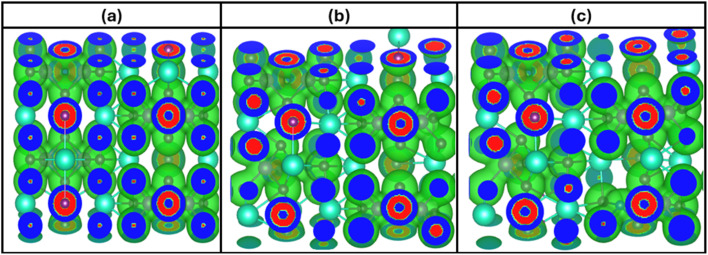
0.10% strained charge density distribtion of Cs_3_Bi_2_I_9_ MIM capacitor materials (a) pure (b) 0.25 mol% Si (c) 0.50 mol% Si.

Effects of mechanical strain on the charge density distribution were also considered as shown in Fig. 15(a–c). Compared to the initial systems (see Fig. 7), there is noticeable increase in charge overlap particularly the doped systems, which is an indication of stronger strain-induced polarization. In the pure system, there is high localization with minimum overlap around Si atoms, indicating some covalent interactions. We also noticed increased overlap on the Bi–I and Si-related regions of the 0.25 mol% Si system. This is because induced strain creates mechanism that breaks symmetry and introduces some localized states. Charge distribution is more distorted in 0.50 mol% Si system. However, proper mechanisms must be adopted to prevent leakage.

#### 3.5.2 Strained piezoelectric coefficients, polarization and dielectric loss tangent (tan *δ*)

Strained piezoelectric coefficient explains how much charges are generated due to mechanical stress. Low values indicate system's low response to mechanical deformation and produce very little charge when compressed, stretched, or bent.^57^ Systems with these properties exhibit poor electrochemical coupling. On the other hand, high values indicate improved charge generation mechanism and sufficient energy conversion efficiency. As shown in Table 7, pure Cs_3_Bi_2_I_9_ reveals negligible piezo electric coefficients, suggesting minimal influence of mechanical strain on charge displacement mechanism, which suppresses its potential as strain-sensitive MIM capacitor material such as sensors. Therefore, it can be regarded as showing low tunability with inefficient electrochemical coupling. Strong out-of-plane piezoelectricity was observed attributable to 0.25 mol% Si-doped system in the *z*-direction, which can enhance charge accumulation and lateral strain, ideal for flexible electronics, strain sensors, and energy harvesting devices. In the 0.50 mol% Si-doped system, results indicate strong vertical polarization due relatively high values of piezoelectric parameters in all directions. This is typical for 0D MIM materials, where vertical charge displacement improves capacitance modulation and strain-tunable dielectric characteristics.

**Table 7:** Piezoelectric coefficients of the strained Cs_3_Bi_2_I_9_ MIM capacitors

MIM systems	*d*_*xx*_ (Cm^−2^)	*d*_*yy*_ (Cm^−2^)	*d*_*zz*_ (Cm^−2^)	Remarks
Pure Cs_3_Bi_2_I_9_	1.00 × 10^−8^	−1.32 × 10^−13^	−4.01 × 10^−16^	Piezoelectric response is negligible
0.25 mol% Si@Cs_3_Bi_2_I_9_	20.00	20.00	50.00	Strong out-of-plane anisotropy and piezoelectricity
0.50 mol% Si@Cs_3_Bi_2_I_9_	40.00	40.00	50.00	Improved out-of-lane piezoelectric response and anisotropy

Data from Table 8 show how 0.10% strain regulates polarization properties of the Cs_3_Bi_2_I_9_ MIM energy storage systems. Polarization in pure Cs_3_Bi_2_I_9_ remains significantly low, aligning with with piezoelectric response already discussed. Findings aligns well with Sun *et al.*,^58^ who identified near-zero polarization because of the centrosymmetric nature and weak ionic displacement. In the 0.25 mol% system, polarization is slighly increased, which is well known for energy storage materials. For the 0.50 mol% system, the induced strain amplified polarization in the *z*-direction because of strain regulated properties.

**Table 8:** Polarization properties of the MIM capacitors under investigation, compared with previous experiments

Systems	Polarization			Remarks	Previos works and reference
	*P*_*xx*_ (Cm^−2^)	*P*_*yy*_ (Cm^−2^)	*P*_*zz*_ (Cm^−2^)		
Pure Cs_3_Bi_2_I_9_	1.33 × 10^−10^	∼0	∼0	Strain intorduced negligible effect	∼0 by Sun *et al.*^58^
0.25 mol% Si@Cs_3_Bi_2_I_9_	1.005	1.005	0.990	Strain slightly increased polarization, remains strong dielectric	0.95 by Adams *et al.*^59^
0.50 mol% Si@Cs_3_Bi_2_I_9_	0.984	0.984	1.005	Anisortropy is more under this strain	0.97 by Adams *et al.*^59^

**Fig. 16:**
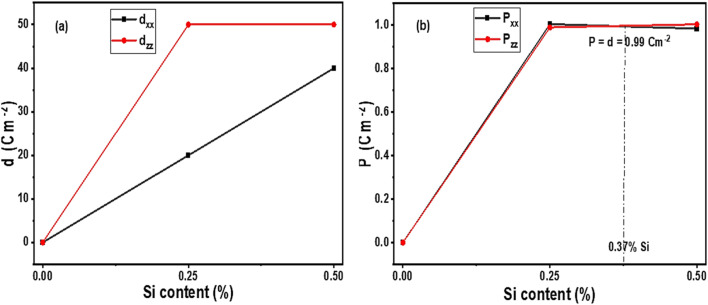
Effects of Si concentration on (a) piezoelectric coefficients (b) polarization, for the Cs_3_Bi_2_I_9_ MIM systems.

To complete this study, it is necessary to address the anticipated leakage of the systems under study. In the context of this work, we consider leakages in terms of the dielectric loss tangent. It is defined as the conduction losses due to leakage currents in the MIM capacitor systems.^60^ High values of tan *δ* signify excess loss while low values result to improved performance due to low conduction losses. We discussed the parameter of tan *δ* based on the relation^61^10
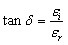
where *ε*_*i*_ and *ε*_*r*_ are the imaginary and real dielectric permittivity respectively. As shown in Table 9, initial tan *δ* is the baseline conductivity loss for the untrained systems. Pure Cs_3_B_2_I_9_ presented significantly lowest loss, while Si doping slightly increases loss due to increased polarization. Introducing 0.10% strain generally lowers loss for pure and 0.25 mol% doped systems, indicating improved performance. The reduced conductivity loss attributable to pure Cs_3_Bi_2_I_9_ is due to low strain-induced polarization, while slightly increased loss in 0.25 mol% is because of increased strain-induced polarization and local asymmetry. More leakage in 0.50 mol% system demonstrates that excessive doping led to more leakage and directional instability. Overall, lowest leakage attributed to pure Cs_3_Bi_2_I_9_ system cannot be considered as achievement because its polarization and piezoelectric properties already indicated its poor ability to generate more charges, which is the reason why leakage is low. We also check some regulatory piezoelectric and polarizing conditions where the systems can perform optimally, with balance between instability and inefficiency. Regarding this, we used piezoelectric and polarization data and plot graphs under various Si concentrations. As shown in Fig. 16(a), at 0.25 mol%, strong piezoelectricity is confirmed, due to uniform piezoelectric values (50 Cm^−2^) in both directions. At 0.50 mol%, *d*_*xx*_ decreases slightly, while *d*_*zz*_ remains high at 50 Cm^−2^, confirming that the stain is on the *z*-direction. According to Fig. 16(b), the balance response in both directions is *d*_*xx*_ = *d*_*zz*_ = 0.99 Cm^−2^, corresponding to Si concentration of 0.75 mol%. At this point, the Si-based Cs_3_Bi_2_I_9_ MIM capacitor will perform with high dielectric constant, strong piezoelectric coupling and better doping stability without excess strain. Therefore, the average condition for improved performance of Si-based Cs_3_Bi_2_I_9_ is when both polarization and piezoelectric coefficients are 0.99, under optimal doping of 0.37 mol% Si.

**Table 9:** Leakage values of the intrinsic and strained Cs_3_Bi_2_I_9_ MIM systems under investigation

MIM systems	Initial tan *δ* (Am^−2^)	Strained tan *δ* (Am^−2^)	Remarks	Obtained results from related works
Pure Cs_3_Bi_2_I_9_	0.20	0.18	Strain slightly reduces dielectric loss, improving efficiency and stability	Sun *et al.* reported improved dielectric behaviour with anisotropic properties under strain^62^
0.25 mol% Si@Cs_3_Bi_2_I_9_	0.22	0.19	Optimal doping: strain lowers loss tangent, enhancing energy storage and reliability	Liu *et al.* showed that strain reduces dielectric loss at 0.25% but failed to provide values^63^
0.50 mol% Si@Cs_3_Bi_2_I_9_	0.24	∼0.3	Excessive Si doping under strain increases dielectric loss, risking leakage and instability	Kastuar and Ekuma mentioned that showed that excessive strain leads to mechanical instability and increased dielectric loss, especially in soft 2D Cs_3_Bi_2_I_3_ structures^64^

Using eqn (9) we have summarized the calculated values of the overall capacitance and leakage parameters of the current systems and then compared with the characteristics of other systems reported. Notably, majority of previous work indicated improvement in the charge characteristics under strain, as shown in Table 10. The obtained leakage parameters were also found to be in good agreement with others. The current result also highlighted significant improvements in the energy storage capacity by the Si-based Cs_3_Bi_2_I_9_ systems than others.

**Table 10:** Capacitance and leakage value of the current systems *vs.* previous works

MIM systems	Initial overall capacitance (µF)	Strain-induced overall capacitance	Leakage range (Am^−2^)	Ref.
Pure Cs_3_Bi_2_I_9_	110.05	110.07; slight increase	0.20–0.18	This work
0.25 mol% Si@Cs_3_Bi_2_I_9_	130.15	140.17; increase	0.22–0.19	This work
0.50 mol% Si@Cs_3_Bi_2_I_9_	130.5	130.38; increase	0.24–0.3	This work
Cs_3_Bi_2_I_9_ (THz dielectric study)	100.90	Slight increase under strain	Negligible leakage	62
Al_2_O_3_/ZrO_2_/SiO_2_ stack	70.40	Tunable with SiO_2_ thickness	0.3	65
BaTiO_3_ (ferroelectric)	100.00	Improved under strain up to 20%	0.10	66

## 4 Conclusions

In this research, energy storage capacity and leakage reduction potential of Si-based Cs_3_Bi_2_I_9_ perovskite were investigated, for use in MIM capacitor applications. All optimizing and energy storage parameters of the various configurations of Si-based Cs_3_Bi_2_I_9_ were determined using DFT procedure and the obtained results were analysed and compared with some previous experimental and theoretical data. Si doping in Cs_3_Bi_2_I_9_ significantly influenced structural, electronic, and dielectric properties relevant to MIM capacitor applications. The incorporation of 0.25 and 0.50 mol% Si reduces unit cell volume due to the smaller ionic radius of Si compared to Bi, while formation energy becomes increasingly negative, indicating enhanced thermodynamic stability—particularly at 0.25 mol%. Band structure analysis reveals Si p′ orbitals introduce states near the CBM, narrowing the band gap and improving band alignment for charge storage. Si atoms act as polarization centres, enhancing dielectric anisotropy and increasing capacitance through higher dielectric constants. Under 0.10% strain, pure Cs_3_Bi_2_I_9_ shows negligible piezoelectric response, whereas Si-doped systems exhibit strong polarization and piezoelectricity, especially in the *z*-direction. Good performance occurs at 0.25 mol%, where improved capacitance and reduced leakage are achieved. Excessive doping (0.50 mol%) introduces directional instability and higher leakage, underscoring the need for controlled doping and strain engineering.

## Conflicts of interest

Authors collectively declare no now conflicts of interests

## Data Availability

All data used in the analysis can be found within this manuscript.
